# Photoperiod sensitivity of Canadian flax cultivars and 5-azacytidine treated early flowering derivative lines

**DOI:** 10.1186/s12870-019-1763-5

**Published:** 2019-05-02

**Authors:** Jia Sun, Lester W. Young, Megan A. House, Ketema Daba, Helen M. Booker

**Affiliations:** 1 Present Address: Department of Plant Science, University of Manitoba, 66 Dafoe Road, Winnipeg, MB R3T 2N2 Canada; 2 Department of Plant Sciences, University of Saskatchewan, 51 Campus Dr, Saskatoon, SK S7N 5A8 Canada

**Keywords:** Photoperiod sensitivity, Flowering time, Flax

## Abstract

**Background:**

Early flowering and maturing flax (*Linum usitatissimum* L*.*) cultivars are better adapted than lines with a longer reproductive phase for the short growing season of the northern Canadian Prairies. We examined the role of long days (LD) and short days (SD) on the time taken to flower in five established flax cultivars and three mutant-derived F_10_ lines. The photoperiod sensitivity of these eight different genotypes was determined using a reciprocal transfer experiment involving weekly transfers between LD and SD environments.

**Results:**

The genotypes tested had varying degrees of photoperiod sensitivity and demonstrated reduced time to flowering if exposed to LD environments prior to a critical time point. The duration of each of the three phases of vegetative growth differed among the genotypes studied. Transfers from SD to LD shortened the vegetative stage, reduced time to flowering, and extended the reproductive phase in the genotypes studied. Mutant-derived lines RE1/2/3 flowered significantly earlier compared to CDC Sorrel, CDC Bethune, Flanders, Prairie Thunder, and Royal. Modelling of the flowering times indicated that transferring the cultivars from SD to LD increased the photoperiod sensitive time; however, different reproductive phases for mutant lines were not defined as parsimonious models were not identified. Expression of the putative flax homologs for *CONSTANS* (*CO*), *FLOWERING LOCUS T* (*FT*), and *GIGANTEA* (*GI*) was examined in the leaves of Royal and RE1/2/3 plants at 10, 15, 19 and 29 days after planting. Expression of putative *FT* homologs was detected in all three early-flowering lines but expression was negligible, or not detected, in Royal.

**Conclusions:**

Models defining the three phases of reproductive development were established for the five cultivars studied; however, it was not possible to identify these phases for the three early flowering and photoperiod insensitive epimutant-derived lines. A putative flax homolog of *FT*, a key regulator of flowering time, is more highly expressed in RE plants, which may condition the day-length insensitivity in the early flowering ‘epimutant’ lines.

**Electronic supplementary material:**

The online version of this article (10.1186/s12870-019-1763-5) contains supplementary material, which is available to authorized users.

## Background

Canada is the largest producer and exporter of flax (*Linum usitatissimum* L.) in the world. However, Canadian flax production is largely limited to the southern parts of Saskatchewan and Manitoba due to a significant risk in the northern part of the grain belt of a killing frost before crop maturity. The development of earlier flowering and, consequently, earlier maturing flax would be beneficial to producers in the areas currently growing flax as well as for expanding the northern limits of flax cultivation in the Canadian Prairies. Traits that increase the suitability of flax to more northerly latitudes include earlier flowering, day-length neutrality (for flower induction), early maturation, frost tolerance, high yield, and suitable oil and protein content [1].

Photoperiod is among the most important exogenous factors that affects flowering time in plants [2]. Photoperiod changes are consistent from year to year and provide a signal to plants of approaching environmental alterations and the requirement to transition from vegetative to reproductive growth [3]. In annual plants, the period from seeding to flowering is divided into three phases [4]: the basic vegetative phase (BVP), known as the “pre-inductive stage” or “juvenile phase”; the photoperiod sensitive phase (PSP), known as the “inductive stage”; and the post-PSP phase (PPP), known as the “post-inductive stage”. A plant grows vegetatively under the most optimal day length during BVP [4, 5].

Cultivated flax (*Linum usitatissimum* L.) is considered a facultative long day (LD) plant, because a short day (SD) photoperiod delays the onset of its reproductive stage [6]. A study that characterized early and late flowering flax genotypes, and conducted under both controlled and field conditions, found that genotypes differed in photoperiod response [7]. However, descriptions of flax responses to photoperiod in the literature are limited [6].

Reciprocal transfer experiments can quantify the vegetative, photoperiod sensitive and reproductive phases of development in different crops. In these experiments, two controlled environment chambers with different settings (e.g., day/night temperature or long/short day length) were employed, with plants transferred between chambers after defined periods. The developmental stages sensitive to the external factors were characterized by observing changes in the induction of the response behavior. For example, in a reciprocal transfer experiment conducted on four rice cultivars (*Oryza sativa* L., SD plant), panicle initiation was delayed by 11–14 days by transfer from SD to LD conditions at both warmer (32/26 °C) and cooler (28/20 °C) day/night temperatures [8]. In soybean (*Glycine max* L*.*), which is SD plant, the maximum difference in days to flowering between LD to SD transfer and SD to LD transfer was 54 days [9]. Finally, in chickpea, a LD plant, flowering time of accessions, including photoperiod sensitive, intermediate, and insensitive accessions, grown under SD was delayed when compared to those under LD [10]. Additional experiments conducted to study photoperiod sensitivity under controlled environments by using reciprocal transfers have been performed with chickpea (*Cicer arietinum* L. [10]), sorghum (*Sorghum bicolor* L. *Moench* [11, 12]), opium poppy (*Papaver somniferum.* L. [13]), chrysanthemum (*Chrysanthemum morifolium* Ramat. [14]) and snapdragon (*Antirrhinum majus* L. [15, 16])

The induction of flower development occurs during the vegetative growth phase that starts after germination. Yin [5] proposed a model, following the model described by Chang et al. [4] in rice, that estimated the duration of each of the BVP, PSP, and PPP phases by observing time to flowering following reciprocal transfers between high and low temperature growth chambers. This approach has allowed researchers to quantify significant environmental impacts on plant growth and development. The present study was conducted to determine if the model described by Yin [5] can be applied to analyze the effect of long/short day photoperiods, as opposed to high and low temperatures, on flower induction in flax and to estimate the length of the BVP, PSP, and PPP. We determined the timing and duration of the photoperiod-sensitive and photoperiod-insensitive phases in selected flax genotypes following a series of reciprocal transfers from LD to SD and vice versa in growth chambers. Additionally, we estimated days to flowering (DTF) under field conditions (equivalent to LD) for CDC Sorrel, Royal, and Royal’s early flowering derivative lines to determine the stability of this trait in the mutant lines.

Flower development is controlled by environmental and genetic factors, as well as the interaction between them [17–19]. Some important genes influencing flowering time include *CONSTANS* (*CO*), *FLOWERING LOCUS T* (*FT*), and *GIGANTEA* (*GI*). These genes belong to various pathways that receive external and endogenous flowering cues, and together contribute to determining optimal timing for the transition from vegetative to reproductive growth. More specifically, *CO* expression is regulated by the circadian clock and red/far-red light ratios, while *GI* expression is affected by the circadian clock and sucrose levels. *GI*, in conjunction with other genes, can regulate *CO* expression while both *GI* and *CO* can influence *FT* expression [17–19]. Overexpression of these foliar expressed genes can lead to an uncoupling of the environmental factors influencing time to flowering, resulting in earlier flowering in *Arabidopsis*. Interestingly, *FT* expression is affected by chromatin remodeling [17], which is a process that can be affected by changes to DNA methylation.

## Methods

Five cultivars (CDC Sorrel, CDC Bethune, Flanders, Prairie Thunder, Royal) and three 5-azacytidine treated mutant lines derived from Royal (RE1, RE2 and RE3) were selected for the reciprocal transfer experiment based on their time to flowering and relative maturity rating [20–25] (Table 1). Flanders, CDC Bethune, and Prairie Thunder are check cultivars utilized in the linseed flax co-operative trials. CDC Sorrel is a relatively new cultivar released in 2005, with a late maturity rating. RE1, RE2, and RE3 are 5-azaC treated early flowering derivative lines of an older flax cultivar, Royal [22], that flower 7 to 13 days earlier than their progenitor [26]. Specifically, approximately 7% of the 5-azaC treated seeds produced plants that flowered earlier than ‘Royal’ did. Three of the earliest flowering selected lines were propagated by single-plant descent to the F_10_ generation to give the RE1, RE2, and RE3 lines used in this study. Early flowering remained consistent in the RE lines through the propagation process. Royal and the RE lines were obtained from Plant Genetic Resources Canada, Agriculture and Agri-Food Canada, Saskatoon. The RE lines are significantly hypomethylated relative to their progenitor [22] and may be referred to as ‘epimutant’ lines, however, it cannot be ruled out that the cause of the early-flowering phenotype is the result of a genetic mutation that occurred during the 5-azacytidine treatment. For the sake of simplicity, RE1/2/3 will be referred to as mutant lines.

**Table 1:** Genotypes used in this project

Genotype	Pedigree	Year of release	Relative maturity description	Variety description
CDC Sorrel	FP956/Vimy	2008	Late	Popularly grown Canadian flax cultivars: CDC Bethune and CDC Sorrel accounted for 25 and 21% of total flax acreage in 2017, respectively (personnel communication, Bert Siemens, Canadian Grain Commission)
CDC Bethune	NorMan/FP857	1998	Medium-Late	
Flanders	McGregor/Dufferin	1989	Late	Check cultivar for maturity in the mid- and long-season zone of the Canadian prairies
Prairie Thunder	FP974 / FP1043	2009	Early	Check cultivar for maturity in the short growing season zone of the Canadian prairies
Royal	–	1939	Medium	Older oilseed variety (1940s)
RE 1	Royal	–	Early	5-azacytidine treated early flowering derivative genotypes
RE 2	Royal	–	Early	
RE 3	Royal	–	Early	

Plants were grown under LD conditions in growth chambers at the University of Saskatchewan at 22 °C/16 °C (12 h/12 h) for a 16 h/8 h photoperiod at a light intensity of 300 μmol photons m^− 2^ s^− 1^ (measured at just above the plant canopy). Plants grown under SD conditions were subject to identical conditions except for a 10 h/14 h photoperiod. Three plants were grown in each pot with 15 pots of each genotype per growth chamber. Starting from the 11th day after seeding, two pots were transferred weekly from one chamber to the other for six weeks, with three pots (nine plants) in each growth chamber used as control pots for each genotype, totaling 120 pots (360 plants) in each chamber. A completely randomized design (CRD) was used for the reciprocal transfer experiment. Pots of each genotype were randomized weekly in each chamber with the entire experiment repeated once. Days to emergence, days to flowering after seeding, number of nodes from bottom of the plant to branching point (NON), plant height (HT), and height from bottom of the plant to the branching point (height to first branch, HTFB) were recorded or measured for each plant. DTF was determined by subtracting days to emergence from days to flowering after seeding. HT and HTFB were measured after flowering, and NON was counted after plants were dried.

Mean flowering time was determined for the six plants from each time point for each genotype. Homogeneity of variances for each round of the experiment was tested using Levene’s test (*p* = 0.05) before combining data from the two replications. CDC Sorrel did not exhibit homogeneity of variance (*p* = 0.05) for the two time replicates, although the data were still combined. The length of each of the sub-phases was quantified using the model proposed by Yin [5] using SAS (University Edition). Significant differences in DTF for the different reciprocal transfers treatments for each genotype were determined using Tukey’s test.

In addition to determining sub-phase lengths using Yin’s model [5], we attempted to identify the position of inflection points between them using hinge regression [10]. A hinge function deals with two linear segments continuously joined together at a hinge [27]. The intersection of the two linear segments, the hinge, is used to identify the changes in slope coefficients and y-intercepts using simultaneous equations for each part of the regression models.

CDC Sorrel, Royal, and mutant lines RE1/2/3 were also evaluated in the field to determine if flowering times were consistent with observations from the growth chambers. Reciprocal crosses were conducted between Royal genotypes (Royal, RE1, RE2, RE3) and CDC Sorrel. The F_1_ generation was grown in a growth chamber at the National Research Council of Canada (Saskatoon) and F_2_ seed harvested from individual F_1_ plants. CDC Sorrel and the early flowering RE1, RE2, and RE3 and the F_2_ and F_3_ Sorrel × Royal/RE1/RE2/RE3 reciprocal crosses were planted in 2012 and 2013 in rows in a type 2 modified augmented design at the Kernen Crop Research Farm (KCRF) in Saskatoon, Saskatchewan, Canada (lat. 52°09′N). The F_3_ plots grown in 2013 were from single plant selections from the F_2_ plots grown in 2012. Planting density was 2.5 g seed in 3.7-m long plots with 0.2-m spacing in 2012 and 1.2-m long plots with 0.2-m spacing in 2013. Within each row, the selection strategy was to tag the 50 earliest-flowering individual plants from each row with the date of flowering. The 2012 growing season comprised 32 check plots and 12 F_2_ plots; the 2013 growing season comprised 229 F3 plots. Planting dates were 2 June 2012 and 31 May 2013. In addition to the single plant selections grown in 2013, F_3_ seed bulked from the F_2_ plots was also grown. A lattice design with three replicates for each genotype was used. These bulk plots were 1.8-m long with six rows, with a plot area of 2.5 m^2^.

All data analysis for modelling and field tests were conducted in SAS 9.3 (SAS Institute) or SAS University Edition using PROC UNIVARIATE, PROC GLM, PROC NLIN, and PROC CORR.

Royal and RE1/2/3 plants were grown in 4 L pots containing #3 Sunshine potting mix, fertilized twice with 500 ml of 15–30-15 supplemented with 0.1 g.L^− 1^ of Cu_2_SO_4_.5H_2_O, and were located in controlled environment growth chambers (18 h / 6 h, 22°/18 °C day/night cycle) at the University of Saskatchewan.

Considerable thought was put into the experimental design for the gene expression study to reduce variability within genotypes, developmental stages, and replicates. At each time point (10, 15, 19 and 29 days after planting) the third leaf of 50 plants was collected from plants having the same developmental indicators (morphologically determined). Leaf tissue was collected at the same time in the photoperiod (after 10 h of illumination) on each collection day. Collected leaves were frozen in liquid nitrogen and stored in an ultralow freezer until use. For each of the three replicates of the three genotypes (RE1, RE2 and RE3), a corresponding set of Royal plants was grown, randomized throughout the growth chamber. Five seeds were sown per pot. Each of the three replicate experiments for each genotype was grown separately and was assigned randomly to one of two available growth chambers. That is, at least 200 RE seeds and 200 Royal seeds (i.e. 50 individuals × 4 time points) were sown into 140 pots and randomly assigned to one of two growth chambers, for each replicate of each Royal versus RE comparison.

Frozen leaf tissue from whole pools was ground under liquid nitrogen and 100–150 mg of powder used for RNA extractions. Total RNA was extracted with a Plant RNeasy Mini Kit (Qiagen), using a final elution volume of 50 μl RNAse-free water. Total RNA was quantified using a nanodrop spectrometer. In a microtiter plate, total RNA (600 ng) from each of the 72 samples (6 ‘genotypes’ (3 RE genotypes and an RC grown alongside each) × 4 time points × 3 replicates) was used as template for cDNA synthesis using a Lunascript Reverse transcriptase kit (NEB).

The *Arabidopsis* mRNA sequences for *CO* (X94937), *FT* (AT1G65480.2) and *GI* (AJ133786) were obtained from The Arabidopsis Information Resource (TAIR; www.arabidopsis.org). Two homologs were identified for each of *CO* (Lus10026909, Lus10020105)*, FT* (Lus10004452, Lus10013532) and *GI* (Lus10028693, Lus10028731) using the *L. usitatissimum* protein database at http://phytozome.jgi.doe.gov. Closer examination suggested that the sequence of Lus10028693 was anomalous and that two possible transcripts were present (see Additional file 1). The sequence of the second transcript appears to be incomplete. Three Taqman probes were designed to amplify the possible transcripts (named Lus10028693.1, Lus10028693.2 and Lus10028731).

The Royal flowering timing gene sequences were obtained from Royal resequencing data (from a different project) using the CDC Bethune genes homologs as the query. Forward and reverse primers (IDT) were designed to complement conserved regions of the two *CO* and *FT* and three *GI* homologs (see Additional file 1). Homolog-specific probes were labelled with FAM. Taqman reactions were performed using 1.0 μl of cDNA, 1 x SsoAdvance Probes Master Mix (BioRad), 300 nM forward and reverse primer, 100 nM probe in a 10.0 μl total volume. The reference gene, glyceraldehyde 3-phosphate dehydrogenase (*GAPDH*)*,* was co-amplified in the same tube for *CO* and *FT*, however the *GI* and reference assays interfered with one another. Accordingly, the three *GI* homologs and *GAPDH* were amplified in separate wells on the same plate. The *GAPDH* amplifications used 100 nM each of forward and reverse primer and Cy5-labelled probe. Thermocycling conditions were: an initial denaturation at 95 °C 2 min, 40 cycles of 95 °C, 10 s; 55–58 °C, 10 s; 72 °C 15 s with a plate read. The qPCR reactions were performed in white 384-well plates using a CFX384 thermocycler (BioRad).

Statistical analyses were performed using RStudio 3.5.1 [28]. Graphing of the RT-qPCR results was performed in Excel. Two-way analysis of variance (ANOVA) were used to determine significant effects of genotype, age, and genotype x age interactions, and Tukey’s test was used for individual pair-wise comparisons.

## Results

### Effect of short day and long day treatments on days to flowering

**Fig. 1:**
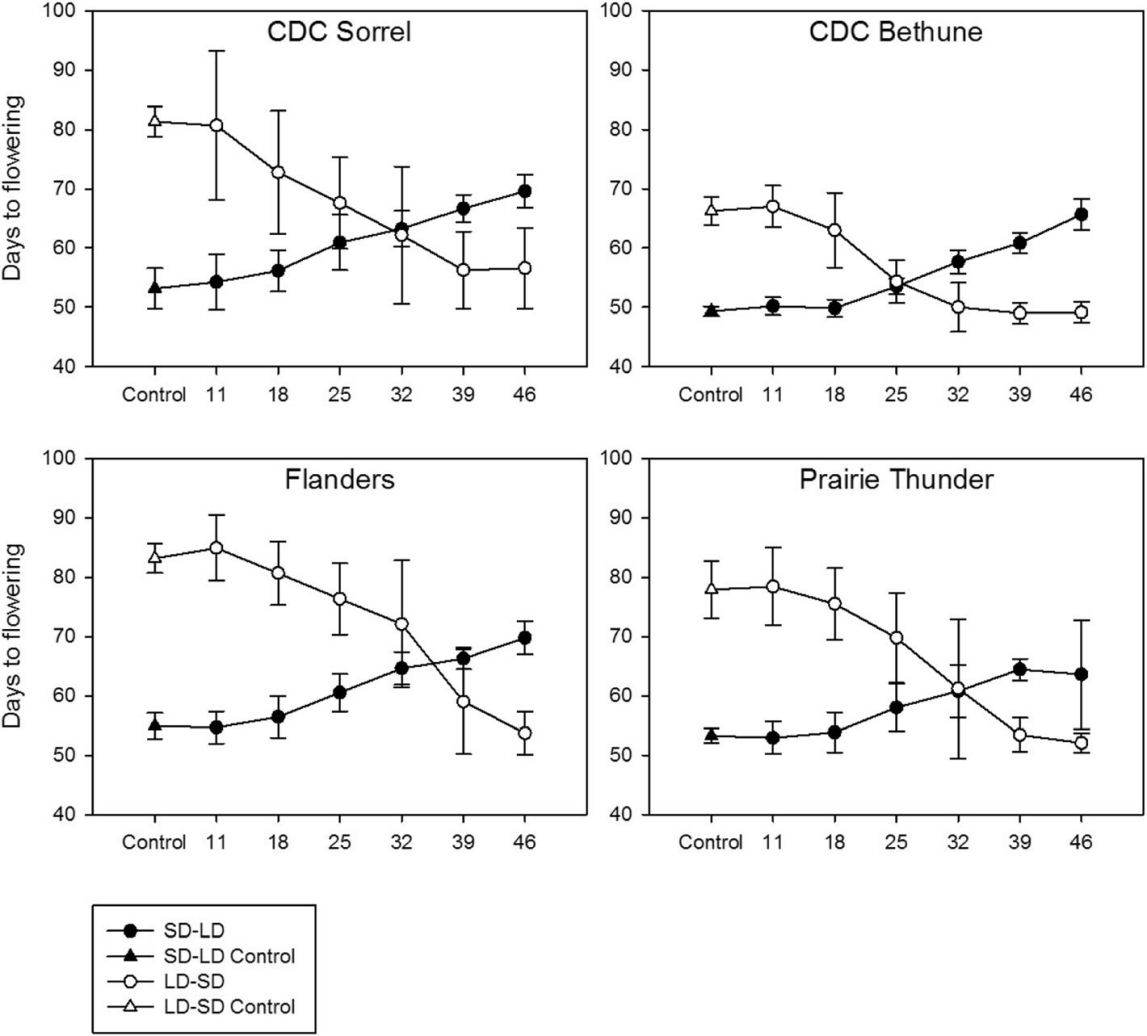
The effect of transferring plants at varying intervals from SD to LD (line with black dots) and LD to SD (line with white dots) on the number of days to first flowering (DTF) for CDC Sorrel, CDC Bethune, Flanders, and Prairie Thunder. Control (CK) plants were not transferred (white and black triangles) and remained in their original chambers. The numbers 11, 18, 25, 32, 39, and 46 refer to the number of days the plants remained in their original chamber before transfer

**Fig. 2:**
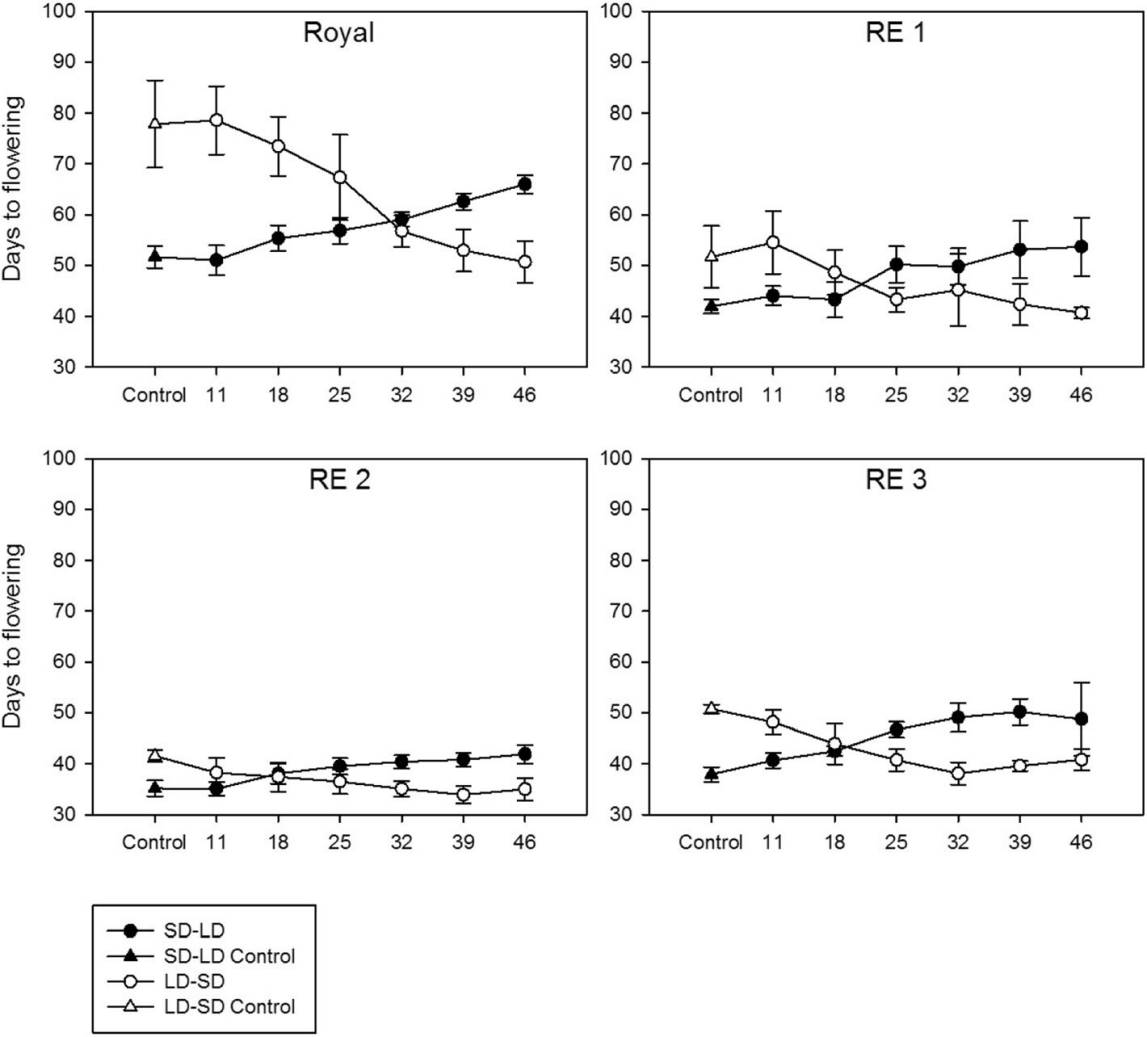
The effect of transferring plants at varying intervals from SD to LD (line with black dots) and LD to SD (line with white dots) on the number of days to first flowering (DTF) for Royal, RE1, RE2, and RE3. Control (CK) plants were not transferred (triangles) and remained in their original chambers. The numbers 11, 18, 25, 32, 39, and 46 refer to the number of days the plants remained in their original chamber before transfer

All of the cultivars used in the experiment showed some degree of photoperiod sensitivity, as indicated by the difference between the DTF of control plants grown in non-inductive SD conditions compared to those under inductive LD (Table 2; triangles in Figs. 1 and 2, also see Additional file 2). Control plants, grown exclusively in SD conditions, had significantly longer DTF than controls grown under LD. Differences between LD and SD DTF were 28, 14, 28, 24, and 24 days for CDC Sorrel, CDC Bethune, Flanders, Prairie Thunder, and Royal, respectively. CDC Bethune appears to be the least photoperiod sensitive cultivar with a DTF of 66 days in SD conditions, compared to 75–83 days for the other cultivars.

**Table 2:** The effect on time to flowering for all genotypes grown under long days (LD) after transferring from short days (SD) to LD (1–6) or LD to SD (1′-6′) environments

Transfer	Genotype			
	CDC Sorrel	CDC Bethune	Flanders	Prairie Thunder
0 (LD CK)	53.2 ^E^	52.0 ^DE^	54.9 ^H^	50.8 ^F^
1	54.3 ^E^	50.2 ^E^	54.7 ^H^	50.6 ^F^
2	56.2 ^DE^	55.0 ^CDE^	56.4 ^GH^	51.8 ^F^
3	60.9 ^CDE^	55.1 ^CDE^	60.5 ^FGH^	55.9 ^EF^
4	63.3 ^BCDE^	57.5 ^CDE^	64.6 ^EFG^	58.5 ^CDE^
5	66.7 ^BCD^	62.4 ^ABC^	66.3 ^EF^	64.3 ^BC^
6	69.6 ^BC^	65.4 ^AB^	69.8 ^CDE^	62.8 ^C^
0′ (SD CK)	81.3 ^A^	66.4 ^AB^	83.2 ^AB^	75.1 ^A^
1′	80.7 ^A^	69.9 ^A^	87.8 ^A^	74.3 ^A^
2′	72.8 ^AB^	67.4 ^A^	80.7 ^ABC^	69.8 ^AB^
3′	67.6 ^BC^	59.4 ^BCD^	76.3 ^BCD^	61.8 ^CD^
4′	62.2 ^BCDE^	52.7 ^DE^	72.1 ^CDE^	52.6 ^EF^
5′	56.3 ^DE^	52.1 ^DE^	59.0 ^FGH^	51.6 ^F^
6′	56.6 ^DE^	51.8 ^DE^	53.8 ^H^	50.3 ^F^
**Transfer**	**Royal**	**RE1**	**RE2**	**RE3**
0 (LD CK)	51.7 ^FG^	41.9 ^DE^	35.2 ^EFG^	37.9 ^F^
1	51.0 ^G^	44.0 ^CDE^	35.1 ^EFG^	40.7 ^DEF^
2	55.3 ^EFG^	43.3 ^CDE^	38.1 ^BCDE^	42.5 ^CDE^
3	56.8 ^EFG^	50.2 ^ABC^	39.5 ^ABCD^	46.7 ^ABC^
4	59.0 ^DEF^	49.8 ^ABC^	40.4 ^ABC^	49.1 ^A^
5	62.6 ^CDE^	53.1 ^A^	40.8 ^AB^	50.2 ^A^
6	66.0 ^CD^	53.7 ^A^	41.9 ^A^	48.8 ^A^
0′ (SD CK)	77.8 ^A^	51.7 ^AB^	41.6 ^A^	50.8 ^A^
1′	78.6 ^A^	54.5 ^A^	38.3 ^BCD^	48.2 ^AB^
2′	73.5 ^AB^	48.6 ^ABCD^	37.4 ^CDEF^	43.9 ^BCD^
3′	67.3 ^BC^	43.3 ^CDE^	36.5 ^DEFG^	40.8 ^DEF^
4′	56.8 ^EFG^	45.2 ^BCDE^	35.1 ^EFG^	38.1 ^EF^
5′	52.9 ^FG^	42. ^ADE^3	33.9 ^G^	39.6 ^DEF^
6′	50.7 ^G^	40.7 ^E^	35.0 ^FG^	40.8 ^DEF^

Transfer 0, CK represents plants not transferred for either LD-CK or SD-CK. Comparisons in time to flowering were conducted using Tukey’s test within genotypes and across transfer times. Highly significant differences within each genotype are indicated with different letters (HSD) (*p* = 0.01)

Differences in DTF between SD and LD controls of RE1, RE2, and RE3 were relatively small compared with the cultivars at 10, 7, and 13 days, respectively (triangles in Fig. 2). Compared to the parental cultivar, Royal, the mutant lines, RE1, RE2, and RE3, flowered 10, 17, and 14 days earlier under LD conditions and 26, 36, and 27 days earlier under SD conditions, respectively (Table 2; Fig. 2). These results indicate all of the mutant lines are less photoperiod sensitive than all of the cultivars, including Royal, and that RE2 was the least sensitive genotype.

Flax is an LD plant, so the greater amount of time spent in LD results in a shorter DTF while longer SD periods result in increased DTF (Table 2; Figs. 1 and 2; Additional file 2). In general, the initial transfer (at 11 days) did not significantly alter DTF as plants were still in the BVP. Transfers during the PSP gradually decreased DTF in LD-SD plants as these individuals’ experienced greater amounts of time in the inductive LD photoperiod as transition dates became later. The opposite was also observed for LD-SD plants. That is, the earlier the transfer from SD to LD conditions, the greater the amount of time the plants spent under inductive photoperiods and the shorter the DTF. Approximately 39–46 days of exposure to LD conditions was sufficient to induce flowering at a similar time to the LD controls for the five cultivars used in this study (Fig. 1). As the plants are photoperiod insensitive during the first 11 days after germination, the minimum amount of time under LD conditions required to induce flowering during the PSP is 28–35 days.

### Modelling effects of photoperiod on the duration of flowering sub-phases

The length of the different sub-phases (BVP, PSP, and PPP) under LD and SD conditions was determined for all eight genotypes (Table 3) [5]. Two alternate models are presented for each of the five cultivars (CDC Sorrel, CDC Bethune, Flanders, Prairie Thunder, and Royal). One model has a higher regression coefficient (normal type) while the other (bold type) was performed with the initial value for the PPP set at zero (meaning undefined) as the PSP extended beyond the last transfer period (46 days) for all SD-LD treated cultivars. These alternative models are bolded in Table 3 and notably have *r* values greater than 0.97. Although the regression coefficients for these models are smaller than those with all variables included are, these fit better with observations of the plants and are more consistent amongst the cultivars.

**Table 3:** Sub-phase length in SD-LD and LD-SD transferred plants

Cultivar or mutant line / r	LD-SD				SD-LD			
	*BVP*	*PSP*	*PPP*	*DTF*	*BVP*	*PSP*	*PPP*	*DTF*
CDC Sorrel / 0.997	8.1	29.0	16.1	*53.2*	6.8	53.9	20.3	81.0
CDC Sorrel / 0.989	**9.3**	**40.1**	**2.8**	52.2	**10.6**	70.7	**0.0**	81.3
CDC Bethune / 0.997	15.9	13.2	19.6	48.7	16.5	29.4	20.6	66.5
CDC Bethune / 0.977	**12.6**	**30.6**	**6.0**	49.2	**12.8**	53.6	**0.0**	66.4
Flanders /0.991	15.2	31.0	9.5	55.7	13.7	62.0	7.9	83.6
Flanders / 0.991	**15.2**	**34.9**	**5.6**	55.7	**13.7**	**69.9**	**0.0**	83.6
Prairie Thunder /0.991	16.0	25.5	10.8	52.3	15.9	51.5	10.3	77.7
Prairie Thunder / 0.987	**14.8**	**33.4**	**4.2**	52.4	**14.7**	**63.0**	**0.0**	77.7
Royal / 0.992	10.5	31.0	9.4	50.9	8.4	60.0	9.0	77.4
Royal / 0.988	**10.5**	**36.8**	**3.6**	50.9	**9.2**	**68.2**	**0.0**	77.4
RE1 / 0.957	9.4	12.5	20.4	42.3	7.5	23.1	22.8	53.4
RE1 / 0.959	12.8	11.7	17.8	42.3	13.3	21.1	19.3	53.7
RE1 / 0.960	16.9	0.7	24.5	42.1	18.0	7.0	29.2	54.2
RE2 / 0.974	6.2	27.3	1.1	34.6	7.2	32.7	1.1	40.9
RE2 / 0.992	10.5	0.5	23.7	34.7	10.2	3.9	27.5	41.6
RE3 / 0.981	5.5	21.4	12.6	39.5	4.2	33.7	12.0	49.9
RE3 / 0.981	10.0	16.9	12.6	39.5	11.3	26.6	12.0	49.9
RE3 / 0.982	13.3	8.8	17.2	39.3	15.6	16.6	18.0	50.2

The length, in days, of the basic vegetative phase (BVP), photosensitive phase (PSP), and post-photosensitive phase (PPP), modeled after Yin [5]. Days to flowering (DTF) was calculated as the sum of the three phases. Two different models are given for each of the cultivars used in this experiment (top half of the table). The first model (normal type) is representative of models with the highest regression coefficient (r). The second model for each cultivar was generated using zero as the initial value for SD-LD PPP (bold type). No single model provided the best fit for the Royal-derived mutant lines RE1, RE2, and RE3 (bottom half of the table), so representative examples are given

The length of the BVP was similar for both models and photoperiod treatments across all cultivars in the experiment (Table 3). Additionally, most of the models for the mutant lines had similar BVP durations. This indicates that vegetative development after germination until the floral induction period is consistent, regardless of genotype or photoperiod. The similar duration of BVP also suggests that the onset of the PSP is similar across cultivars.

The main differences between models and genotypes were with respect to the length of the PSP. The duration of the PSP in SD-LD plants was twice that of the LD-SD plants within a cultivar for a model (Table 3). In SD-LD plants, where the initial value of the PSP is set to zero (meaning PPP is undefined), the end of this phase coincides with the start of flowering. In models in which the initial estimation of PPP is non-zero, the transition from PSP to PPP occurs after the final transfer point in this experiment and therefore would not have been observed in these results. Additional transfers would be required to observe this transition in the cultivars examined. It is not possible to determine if there is a transition from PSP to PPP in SD-LD plants due to this limitation in the experimental design. CDC Bethune comes the closest to having a transition from PSP to PPP during the transfers with a cumulative duration of BVP and PSP of 49.2 days, close to the day of the final transfer (46 days after planting).

Multiple models with similar regression coefficients were developed for RE1, RE2, and RE3, some of which are shown in Table 3. Determining which model is most correct is not possible. The difficulty in identifying a parsimonious model may be due to the uniform DTF of the SD-LD and LD-SD plants and/or because some phases are very short (shown, for example, in some of the RE2 models). Together, these observations suggest that Yin’s work [5] cannot be used to model flower development in photoperiod insensitive genotypes.

An attempt was made to identify the transition points between BVP, PSP, and PPP using hinge regression, after Daba et al. [10]. This analysis was unable to determine definitively the inflection points between phases using the data generated by this experiment (data not shown). The small number of time points used in the experiment may be one reason why this post hoc analysis was unsuccessful. Application and use of Hinge hyperplane for regression is discussed in [27]. The model used is extensively employed in multivariate regression and classification. Daba et al. [10] identified three phases in chickpea using this technique but also underlined the drawbacks of the model. Judiciously deciding a good initial starting point is important because variables in the first hinge could be selected almost by coincidence. We tried to apply this technique to augment our findings, but the limited number of time points in our data restricted our attempt to utilize the model for photoperiod phase identification.

### Developmental comparisons among different genotypes

Correlations among the developmental characteristics DTF, NON, HT, and HTFB evaluated in CDC Bethune, CDC Sorrel, Flanders, and Prairie Thunder were highly significantly (Table 4; Additional file 2). Similar results were observed for RE2 and RE3. Exceptions to the high degree of correlation observed between traits assessed were HT and DTF or NON for Royal and HT and DTF for RE1 (Table 4).

**Table 4:** Correlations between traits of days to flowering (DTF), number of nodes (NON), plant height (HT), and height to first branch (HTFB) for all genotypes

Genotype	Trait	DTF	NON	HT	HTFB
CDC Sorrel	DTF	1.000			
	NON	0.822***	1.000		
	HT	0.605***	0.633***	1.000	
	HTFB	0.629***	0.667***	0.818***	1.000
CDC Bethune	DTF	1.000			
	NON	0.864***	1.000		
	HT	0.347***	0.388***	1.000	
	HTFB	0.624***	0.640***	0.671***	1.000
Flanders	DTF	1.000			
	NON	0.889***	1.000		
	HT	0.556***	0.702***	1.000	
	HTFB	0.743***	0.806***	0.807***	1.000
Prairie Thunder	DTF	1.000			
	NON	0.868***	1.000		
	HT	0.481***	0.754***	1.000	
	HTFB	0.624***	0.844***	0.929***	1.000
Royal	DTF	1.000			
	NON	0.883***	1.000		
	HT	−0.005	0.117	1.000	
	HTFB	0.648***	0.690***	0.162*	1.000
RE1	DTF	1.000			
	NON	0.740***	1.000		
	HT	0.150	0.164*	1.000	
	HTFB	0.568***	0.673***	0.409***	1.000
RE2	DTF	1.000			
	NON	0.612***	1.000		
	HT	0.462***	0.332***	1.000	
	HTFB	0.500***	0.653***	0.475***	1.000
RE3	DTF	1.000			
	NON	0.743***	1.000		
	HT	0.300***	0.158*	1.000	
	HTFB	0.774***	0.732***	0.384***	1.000

Correlation tests conducted within each genotype; *** indicates significant level at *p <* 0.001, and * for *p <* 0.05

### Performance under field conditions

**Fig. 3:**
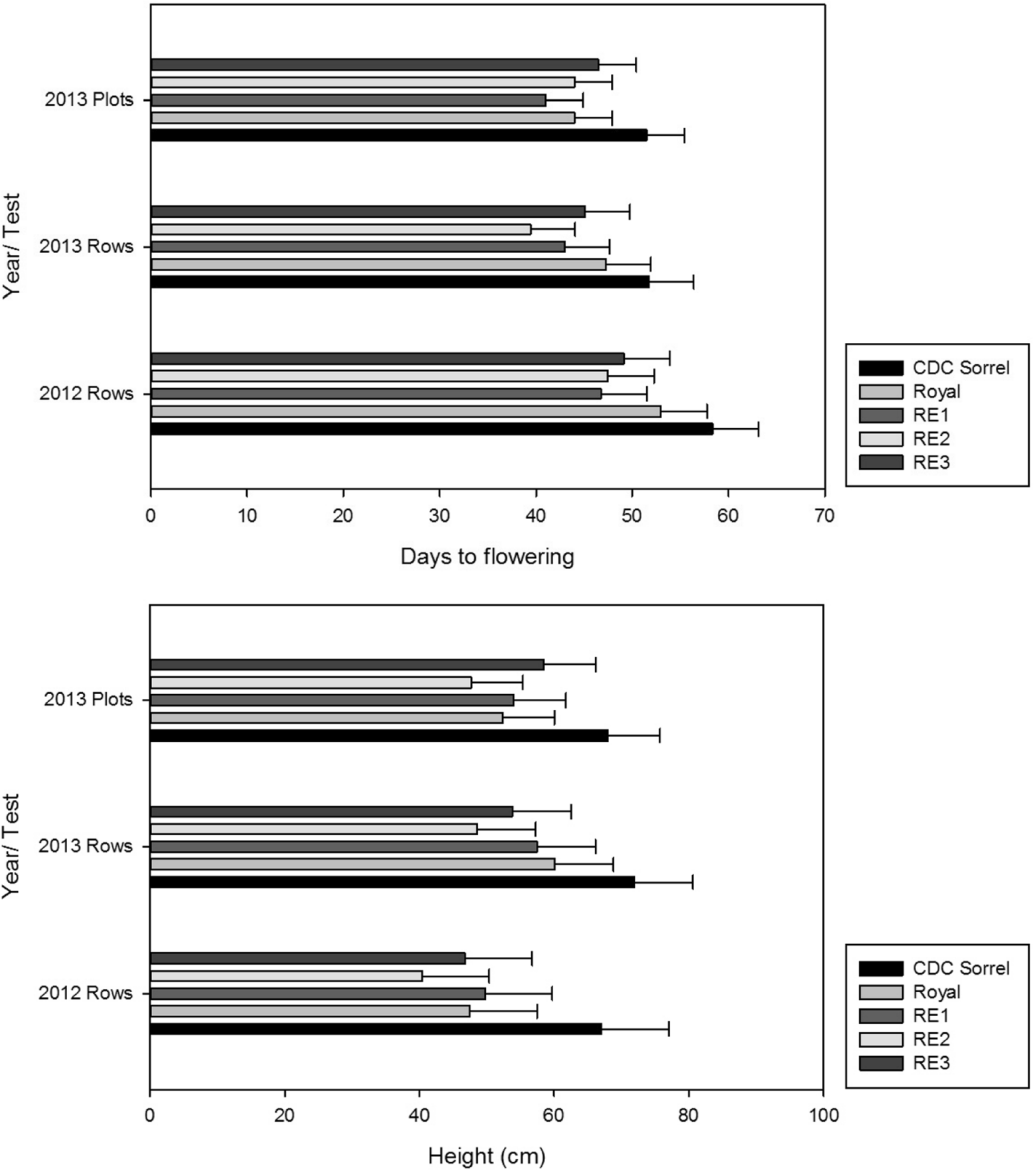
Comparisons of days to flowering and plant height among CDC Sorrel, RE1, RE2, and RE3 grown under field conditions

In 2012 and 2013, CDC Sorrel was significantly taller and later flowering than Royal, RE1, RE2, and RE3 (Fig. 3; Additional file 3). In the 2012 single-row F_2_ population, CDC Sorrel flowered 58 days after seeding, while Royal, RE1, RE2, and RE3 flowered 53, 47, 48, and 49 days after seeding, respectively. In the 2013 single-row F_2_ population, Royal, RE1, RE2, and RE3 flowered 47, 43, 40, 45 days after seeding, all of which were earlier than CDC Sorrel was (52 days). RE2 was the earliest flowering genotype. This was consistent with that observed in the 2013 F_3_ bulk population, in which Royal, RE1, RE2, and RE3 all flowered significantly earlier than CDC Sorrel (52 days) and, among the RE lines, RE2 flowered 41 days after seeding, which was significantly earlier than Royal (45 days) (LSD = 1.79; *p ≤* 0.05).

The 2013 single-row population parental genotypes CDC Sorrel, Royal, RE1, RE2, and RE3 had heights of 72, 60, 57, 49, and 54 cm, respectively (Fig. 3). The 2013 F_3_ bulk population parental genotypes CDC Sorrel, Royal, RE1, RE2, and RE3 had heights of 68, 50, 53, 47, and 57 cm, respectively, with Royal, RE1, RE2, and RE3 all significantly shorter than CDC Sorrel (LSD = 6.41; *p ≤* 0.05).

### Expression of *FT*, *CO*, and *GI* in leaves of 10, 15, 19 and 29 day old ‘Royal’ and RE1/2/3

**Fig. 4:**
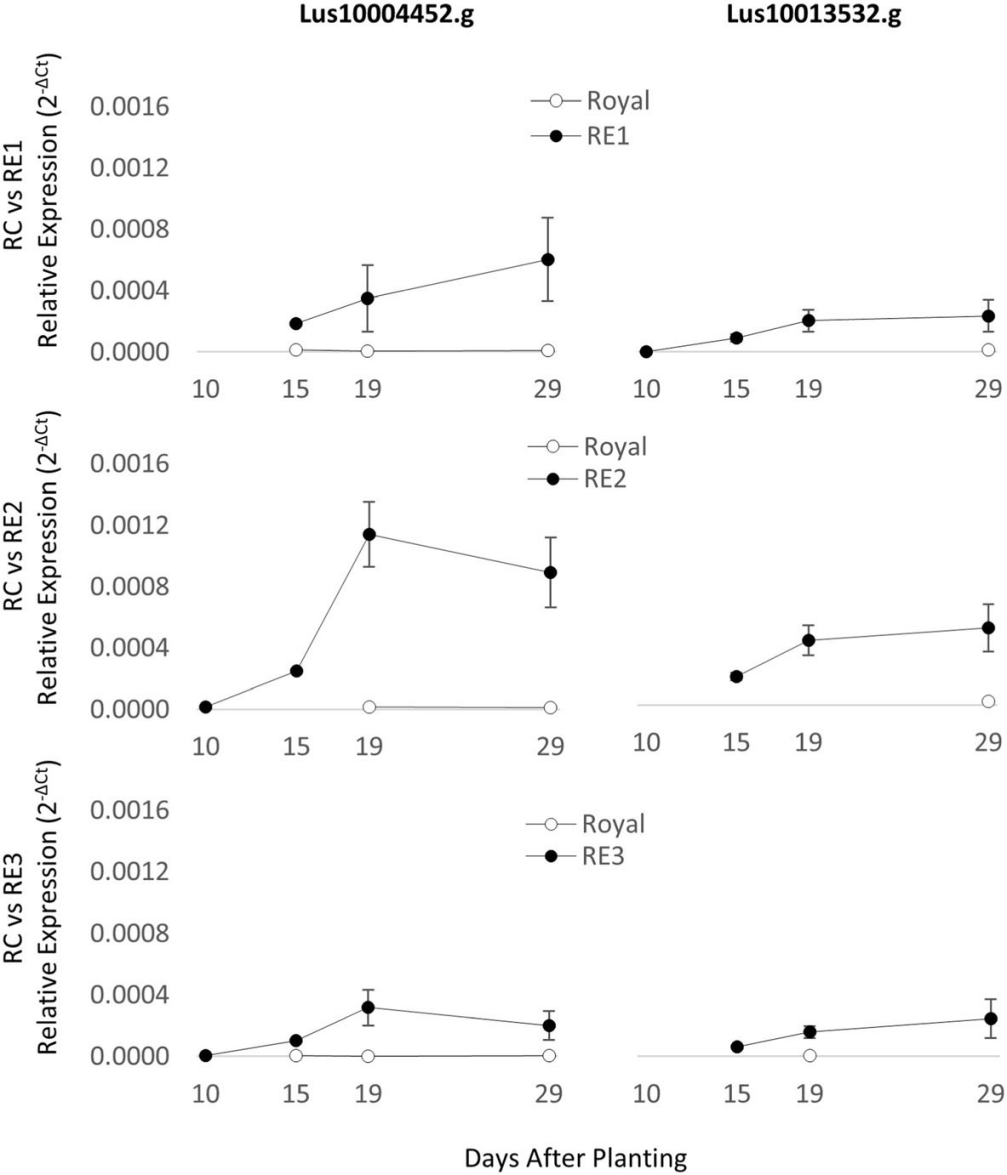
Relative expression of *FT* homologs Lus10004452 and Lus10013532 in RE1/2/3 and 'Royal' leaves. RT-qPCR assays were performed in triplicate on cDNA from total RNA extracted from the third leaf, 10, 15, 19 and 29 days after planting. Expression of Lus10004452 and Lus10013532 in Royal was negligible or was observed very late in the amplification. Three replications of the experiment were performed for each genotype. Error bars show standard errors

Expression of both putative *FT* homologs (Lus10004452 and Lus10013532) in RE1/2/3 was detected in all genotypes (Fig. 4; Additional file 1). A significant effect of genotype on expression was observed, relative to ‘Royal’, in both RE2 (*p* < 0.01) and RE3 (*p* < 0.05), with higher transcript abundance being observed in the early-flowering lines (Fig. 4). The expression of Lus10004452 in RE2 increased significantly over days 15–19 (*p* < 0.05) and plateaued by day 29, and a similar trend was observed for both Lus10004452 and Lus10013532 in all three RE genotypes. In comparison, expression of the same homologs was negligible or undetectable in Royal over the duration of the experiment. Unexpectedly, expression of the putative *CO* (Lus10026909 and Lus10020105) and *GI* (Lus10028693.1, Lus10028693.2 and Lus10028731) homologs were not significantly different between RE1/2/3 and Royal. Expression of all *CO* homologs, and *GI 1.2*, differed significantly across time points for all RE versus Royal comparisons (see Additional file 1). Age also had a significant effect on expression for *GI 1.1* in the RE2 x RC contrast.

## Discussion

This study, and others, demonstrates that flax is an LD plant and that DTF is impacted by different photoperiods (LD versus SD) [6, 7, 29]. In all genotypes, control plants grown under LD had significantly shorter DTF than those under SD, exhibited by a delay in flowering under SD and/or accelerated under LD. The smaller difference in DTF for LD and SD CDC Bethune control plants is interesting and may account for the widespread distribution of this cultivar across the Canadian Prairies. Moreover, transfers from LD to SD during PSP influenced DTF for all genotypes, and vice versa, although transfers during the photoperiod insensitive phases (BVP and PPP) did not alter the DTF for most genotypes.

The response to photoperiod varied between genotypes. For instance, the duration of the PSP varied considerably amongst the cultivars used in this experiment (Table 3); however, this phase was non-existent and/or could not be modelled in the photoperiod insensitive mutant lines. The magnitude of the influence of photoperiod on DTF differed among the genotypes studied. For example, RE2 was the least photoperiod sensitive genotype followed by RE3, RE1, Flanders, CDC Bethune, Royal, Prairie Thunder, and CDC Sorrel, respectively (Table 2).

In the early flowering lines (RE1, RE2, and RE3), a reduction in photoperiod sensitivity was associated with an earlier flowering phenotype under LD and SD controlled environments (Table 2; Figs. 1 and 2) as well as in field tests (Fig. 3). Thus, characterizing the RE lines as day-length neutral is reasonable.

Flax is the last crop to mature on the Canadian Prairies and an earlier flowering phenotype is an important breeding target. Reduced time to flowering and maturity are also major goals for other crops in the northern Prairies, including Ethiopian mustard (*Brassica carinata*; [30]) and chickpea [31]. Indeterminate crops such as chickpea, lentil (*Lens culinaris* Medik), and canola (*Brassica juncea* L.) have been improved by breeding for earlier flowering and/or a longer reproductive duration [30, 32].

Traits associated with better yield production in flax grown in the northern Prairies include lines with earlier flowering (these might be daylength insensitive lines that flower early regardless of day length, or daylength sensitive lines that flower earlier under long days) that triggers earlier maturity. Importantly, RE1,2,3 derivative lines of ‘Royal’ flax represent new sources of variation for flowering time. This study for the first time lays the foundation for mining (epi)allelic variation in breeding for flax improvement.

Traits correlated with DTF in this study included NON, HT, and HTFB. Plant HT and HTFB were reduced in plants grown under LD compared with SD, suggesting that growth under LD extends the reproductive phase in flax through early flowering that shortens the vegetative stage; these observations are corroborated by Fieldes and Harvey [33]. Early flowering limits vegetative growth, enables reproductive growth to occur before terminal stress, and usually correlates with early maturity [34]. Early flowering in chickpea (*Cicer arietinum*) leads to earlier maturity by 10 days [31].

The four developmental traits (DTF, NON, HT, and HTFB) were significantly correlated with each other in CDC Sorrel, CDC Bethune, Prairie Thunder, and Flanders but not in Royal and RE1. In Royal and RE1, RE2, and RE3, NON and HTFB were significantly correlated with DTF. These results are supported in part by Fieldes and Harvey’s [33] study that suggests node/leaf number is a useful indicator for predicting DTF in flax [33]. Our results also show that NON and HTFB could be used as indications for predicting flowering time in flax.

Compared with the day-neutral RE lines, results for the cultivars CDC Sorrel, CDC Bethune, Flanders, and Prairie Thunder show that plants exposed earlier to a favorable day-length environment adjust earlier and initiate the reproductive stage of development.

All genotypes examined in this study had flowering times different from one another and large differences were observed in the duration of their photoperiod sensitive phases. The earlier flowering RE lines were significantly less photoperiod sensitive than their progenitor Royal. The model developed by Yin [5] can be applied satisfactorily to flax cultivars; however, it did not function well for the photoperiod insensitive RE mutant lines. Field tests confirmed that the early flowering trait in RE1/2/3 was stable under field conditions, with flowering occurring significantly earlier than the cultivar CDC Sorrel.

Expression of genes involved in determining flowering timing was determined in leaves as photoperiod is detected in these organs [17–19]. For most time points expression of the *CO* and *GI* homologs was not significantly different between RE1/2/3 and Royal, but *FT* was clearly expressed in the former lines, albeit at a low level, and not in the latter (Fig. 4 and Additional file 1). These results are surprising as both *CO* and *GI* expression are central to the photoperiod induction pathway upstream of *FT* [17–19]*.* The shared expression patterns of these genes in the early lines and their progenitor line may indicate that altered expression of *CO* and *GI* is not involved in determining early-flowering in RE1/2/3. Alternately, in the case of *CO*, which is a member of a large gene family, it is possible that a *CO-like* gene has been targeted and that further attempts to amplify the true homolog of *CO* in flax will acknowledge a role of CO in the early-flowering phenotype of the mutant lines. Our observation of earlier, and higher *FT* expression corresponds with earlier flowering in RE1/2/3 and the apparent photoperiod insensitivity in these lines. One speculation is that 5-azacytidine-induced changes in DNA methylation changed the chromatin structure of a *cis*- or *trans*-acting regulatory element of *FT*, resulting in higher, or earlier, expression of this gene in the RE1/2/3 lines and consequently, loss of photoperiod sensitivity. Alternatively, overexpression of an *FT*-inducing gene, or inhibition of an *FT*-repressing gene, could be leading to greater *FT* expression [17, 18]. The *FT*-influencing genes in RE1/2/3 may not necessarily lie in the photoperiod induction pathway, since *FT* is a hub for inputs from multiple pathways, further complicating identification of the (epi)mutations. That is, overexpression of *FT* may be due to changes in the autonomous induction pathway, or alterations in the circadian clock. An examination of the leaf transcriptome could shed light on the mechanism of increased *FT* expression, differences in *FT* regulation, and other pleiotropic effects between the three RE genotypes.

## Conclusion

Previous research shows that treatment with 5-azaC alters DNA methylation patterns in flax and results in earlier flowering in the oilseed variety Royal [22]. This study demonstrates for the first time using short day–long day transfer studies that the early flowering derivative RE lines are significantly less photosensitive than the Royal progenitor genotype. Moreover, the early flowering lines expressed *FLOWERING LOCUS T* in leaves, whereas Royal did not from 15 to 29 days after planting (corresponds to the vegetative period in Royal). To determine the regions of the flax genome associated with the early flowering characteristic in the RE lines mapping populations derived from crosses of Royal and RE lines are being studied. Furthermore, to better elucidate the underlying genetic determinants of the early flowering in the mutant lines we are examining the leaf transcriptome.

## Additional files

Additional file 1: Flowering gene expression study. Information on putative flax homologs, primer sequences, homolog alignments and expression levels is provided. (DOCX 669 kb)

Additional file 2: Data from reciprocal cross experiments. Flowering time, number of nodes, plant height and branching height for the five cultivars and three mutants exposed to different short-day and long-day durations is given. (XLSX 81 kb)

Additional file 3: CDC Sorrel x (Royal or RE1/2/3) field data. Start of flowering time, start of full flowering, days to maturity and plant height for F2, F3 and bulk F3 CDC Sorrel x RE1/2/3 or Royal populations. (XLS 81 kb)

## Abbreviations

ANOVA: Analysis of variance
BVP: Basic vegetative phase
*CO*: *CONSTANS*
CRD: Completely randomized design
DTF: Days to flowering
*FT*: *FLOWERING LOCUS T*
GAPDH: Glyceraldehyde 3-phosphate dehydrogenase
*GI*: *GIGANTEA* (*GI*)
HT: Plant height
HTFB: Height to first branch
LD: Long day
NON: Node number
PPP: Post-photoperiod sensitive phase
PSP: Photoperiod sensitive phase
SD: Short day
TAIR: The Arabidopsis Information Resource
