# Supplementary material for: Photoperiod sensitivity of Canadian flax cultivars and 5-azacytidine treated early flowering derivative lines
Source: BMC Plant Biol. 2019 May 2;19:177. doi: 10.1186/s12870-019-1763-5 (PMC6498464; doi:10.1186/s12870-019-1763-5)
Supplement: Supplementary file 1 — Flowering gene expression study. Information on putative flax homologs, primer sequences, homolog alignments and expression levels is provided. (DOCX 669 kb) [file 12870_2019_1763_MOESM1_ESM.docx]

Additional file 1

Table S1: Flowering timing genes used in this study

| ***Arabidopsis*** gene | Putative flax homologs | Nickname | Protein identity%/ similarity% (Blosum62) | Pseudomolecule coordinates (LG:bp) |
| --- | --- | --- | --- | --- |
| X94937 | Lus10026909 | CO1 | 42.1 / 69.3 | 13:2630788-2631934 |
|  | Lus10020105 | CO2 | 42.6 / 69.3 | 2:3825615-3826764 |
| AT1G65480.2 | Lus10004452 | FT1 | 73.7 / 92 | 4:7158300-7161800 |
|  | Lus10013532 | FT2 | 73.7 / 92 | 13:7519400-7522200 |
| AJ133786 | Lus10028693.1 | GI1.1 | 73.5 / 87.8 | 14: 1519631-1514240 |
|  | Lus10028693.2 | GI1.2 | 72.9 / 88.2 | 14:1513079-1509930 |
|  | Lus10028731 | GI2 | 69.6 / 84.1 | 14:1748717-1754273 |

Table S2: qPCR primers and probes used in this study

| Gene | Primer / probe name | Sequence (+ fluorophore) | Tm (°C) |
| --- | --- | --- | --- |
| CO | LuCO-fwd | TTGAAGCGCAGGAGCAG |  |
|  | LuCO-rev | AGATACGCTGTGGCTCAAG |  |
|  | LuCO1-pr | AGGCTCCGGTCATGATGAATGACCACTG (FAM) |  |
|  | LuCO2-pr | TGCTCCCGTCATGAATATGAATGACCAC (FAM) |  |
| FT | LuFT-fwd | ACCAAGTCCTAGCGATCCTA |  |
|  | LuFT-rev | GTCTCTCGTTGGCAGTTAAA |  |
|  | LuFT1-pr | AACTCTACAACTTAGGTTCCCCCGTTG (FAM) |  |
|  | LuFT2-pr | AACTCTACAACTTAGGTCCGCCTGTTG (FAM) |  |
| GI | LuGI-fwd | CTTGTTTCACAGGTATTATGC |  |
|  | LuGI-rev | GTATGTACAAGTTCCATGACA |  |
|  | LuGI1.1-pr | CTCTACTCTTCCGCATCCTGTCA (FAM) |  |
|  | LuGI1.2-pr | TGATGGAGTTGAAGTACAGCATGAACC (FAM) |  |
|  | LuGI2-pr | CACTACGCCAAGTTGATTGCATCG (FAM) |  |
| GAPDH | GAPDH-fwd |  |  |
|  | GAPDH-rev |  |  |
|  | GAPDH-probe | (Cy5) |  |

Supplementary figure: Flowering timing gene alignment and primer/probe alignment


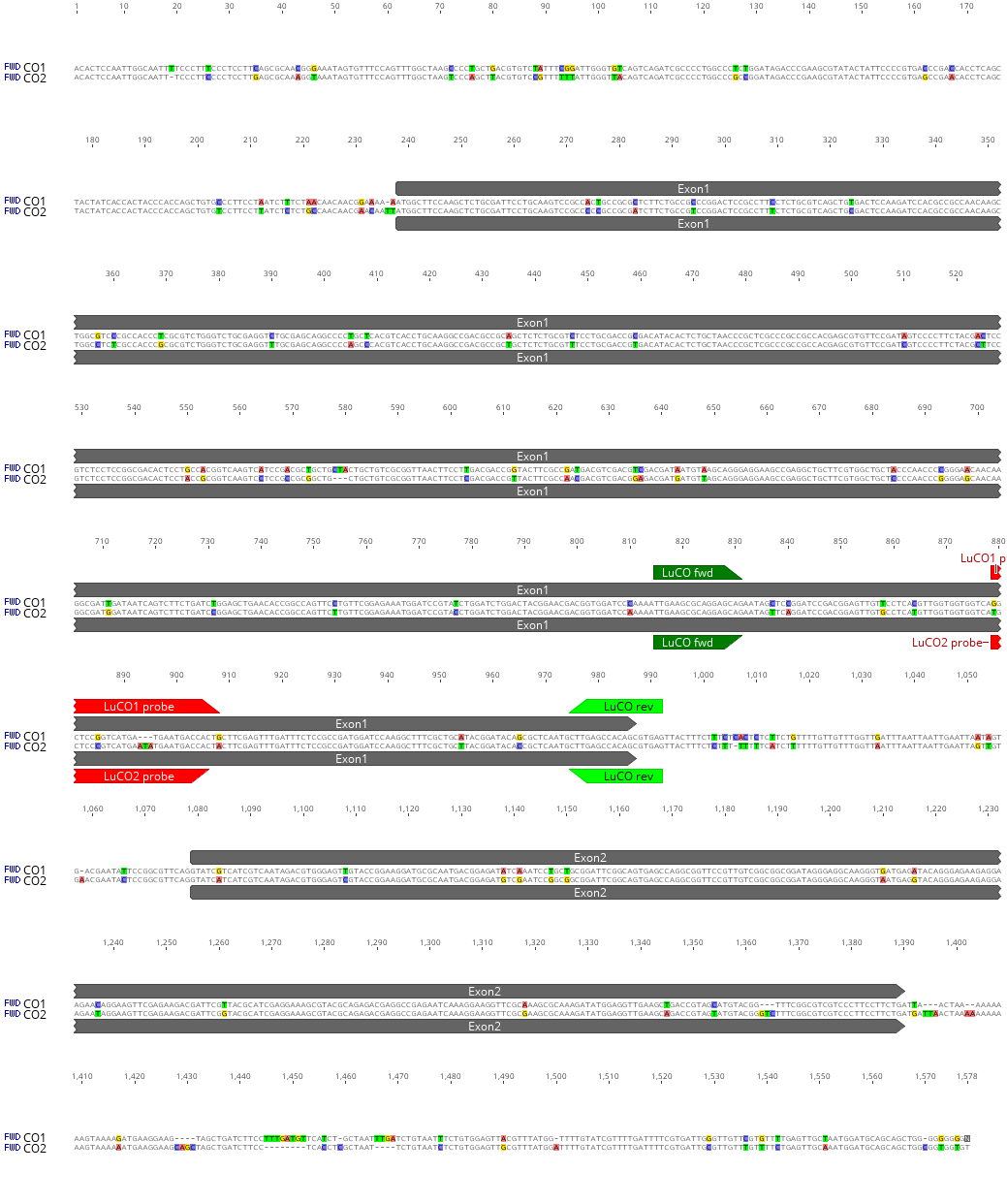


Royal CO1 and CO2 alignment. Exons (grey bars), primers (green bars) and homolog specific probes (red bars) are shown. Mismatched nucleotides are highlighted in colour.


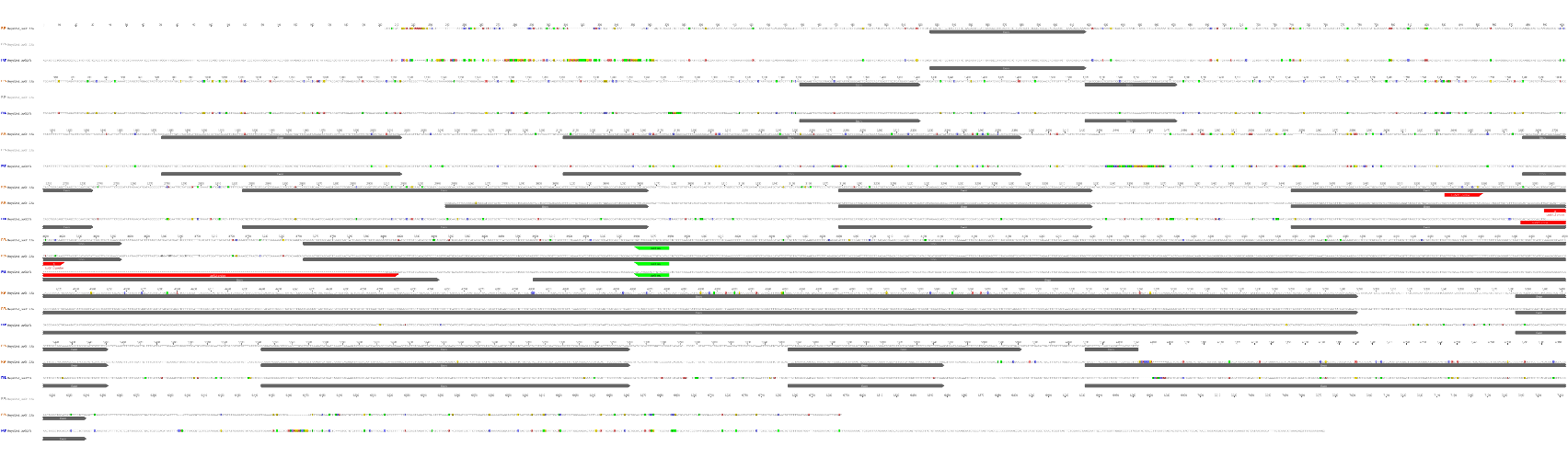


Royal GI1.1, GI1.2 and GI2 alignments. Note, forward primer overlaps the junction between two exons and is not shown in this diagram. In addition, the probe for GI2 overlaps a space inserted into the exon for alignment purposes. Exons (grey bars), primers (green bars) and homolog specific probes (red bars) are shown. Mismatched nucleotides are highlighted in colour.


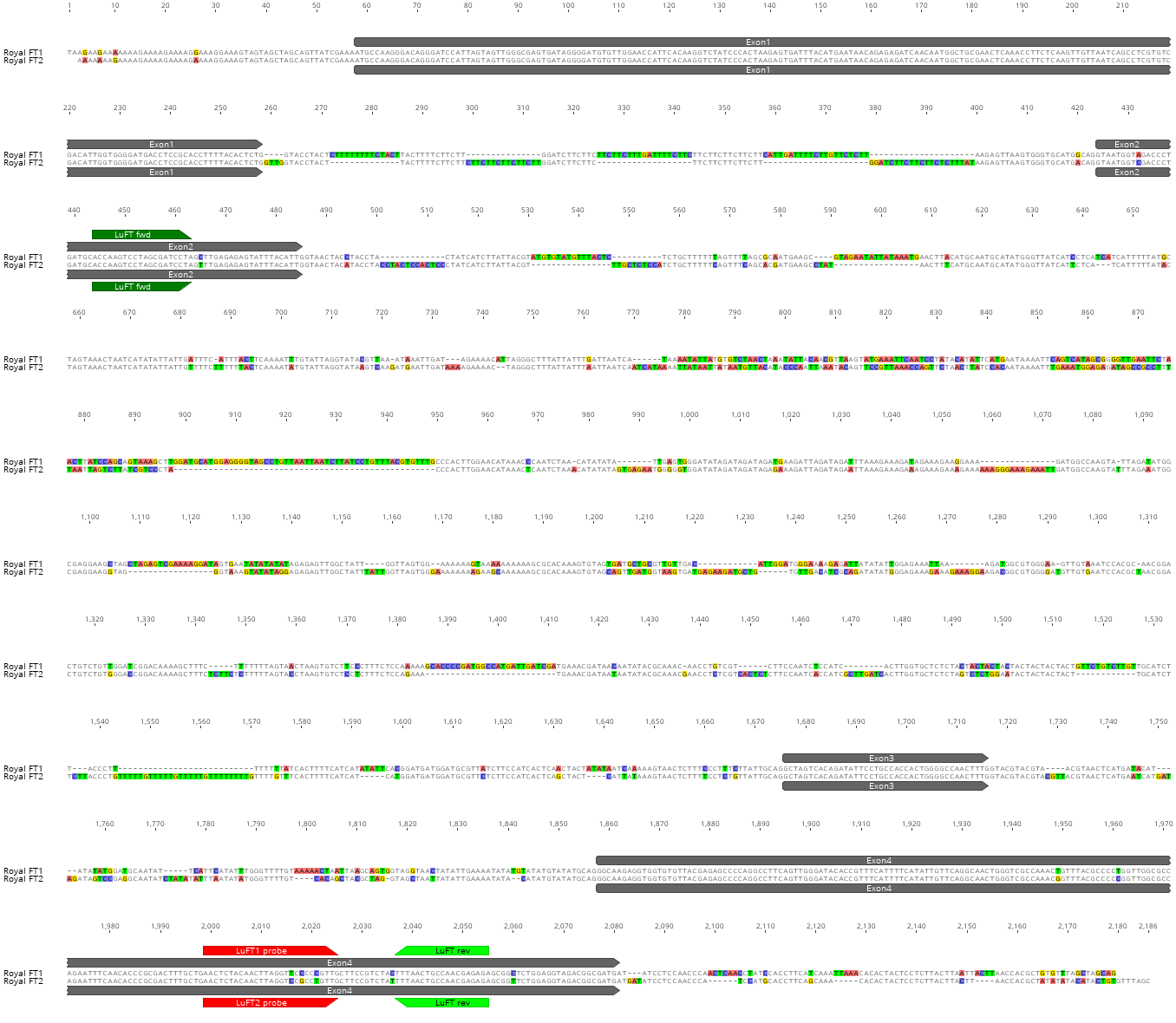


Royal FT1 and FT2 alignment. Exons (grey bars), primers (green bars) and homolog specific probes (red bars) are shown. Mismatched nucleotides are highlighted in colour


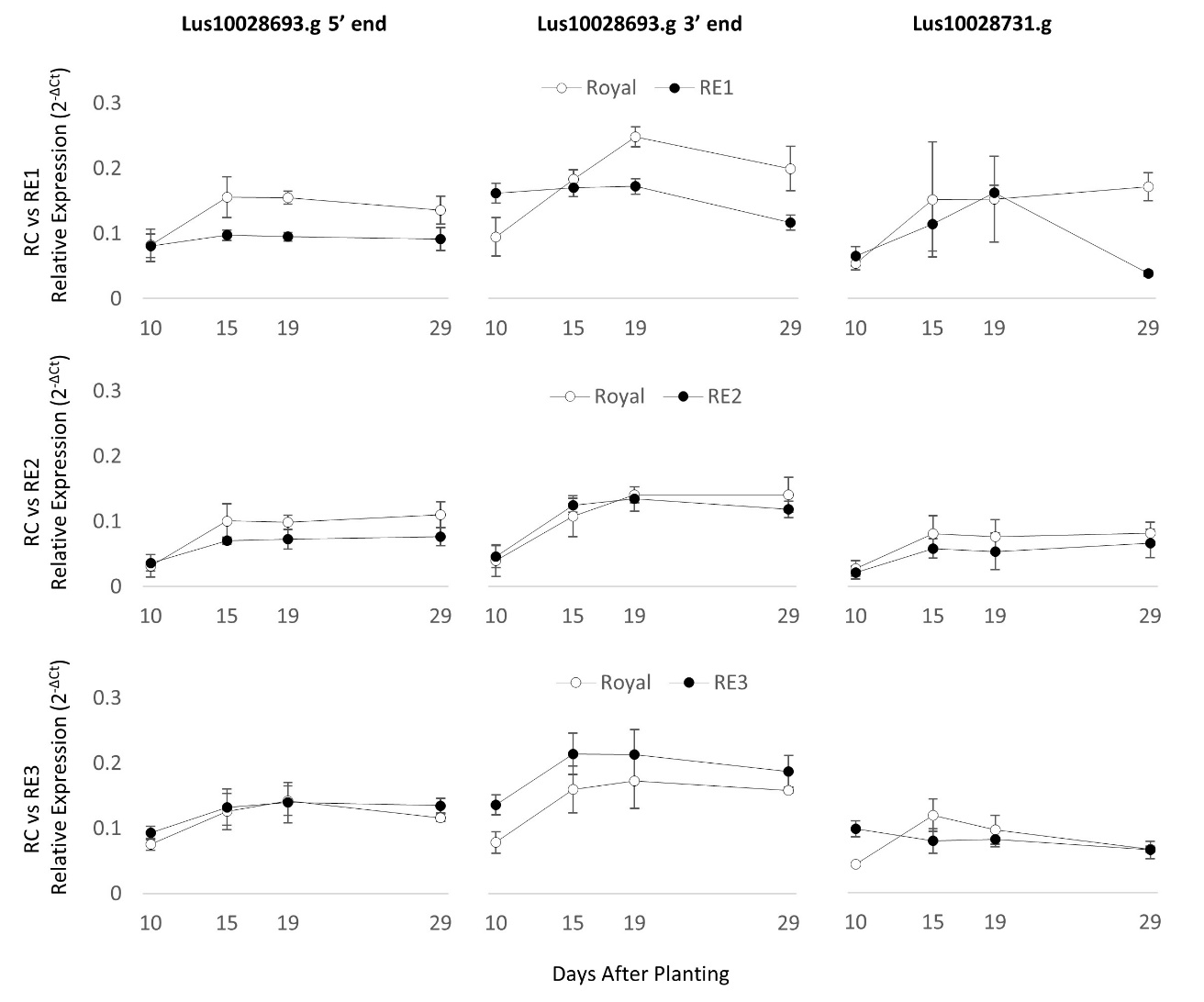


Relative expression of *GI* homologs Lus10028693.1, Lus10028693.2 and Lus10028731 in RE1/2/3 and Royal leaves. RT-qPCR assays were performed in triplicate on cDNA from total RNA extracted from the third leaf, 10, 15, 19 and 29 days after planting. Three replications of the experiment were performed for each genotype. Error bars show standard errors.


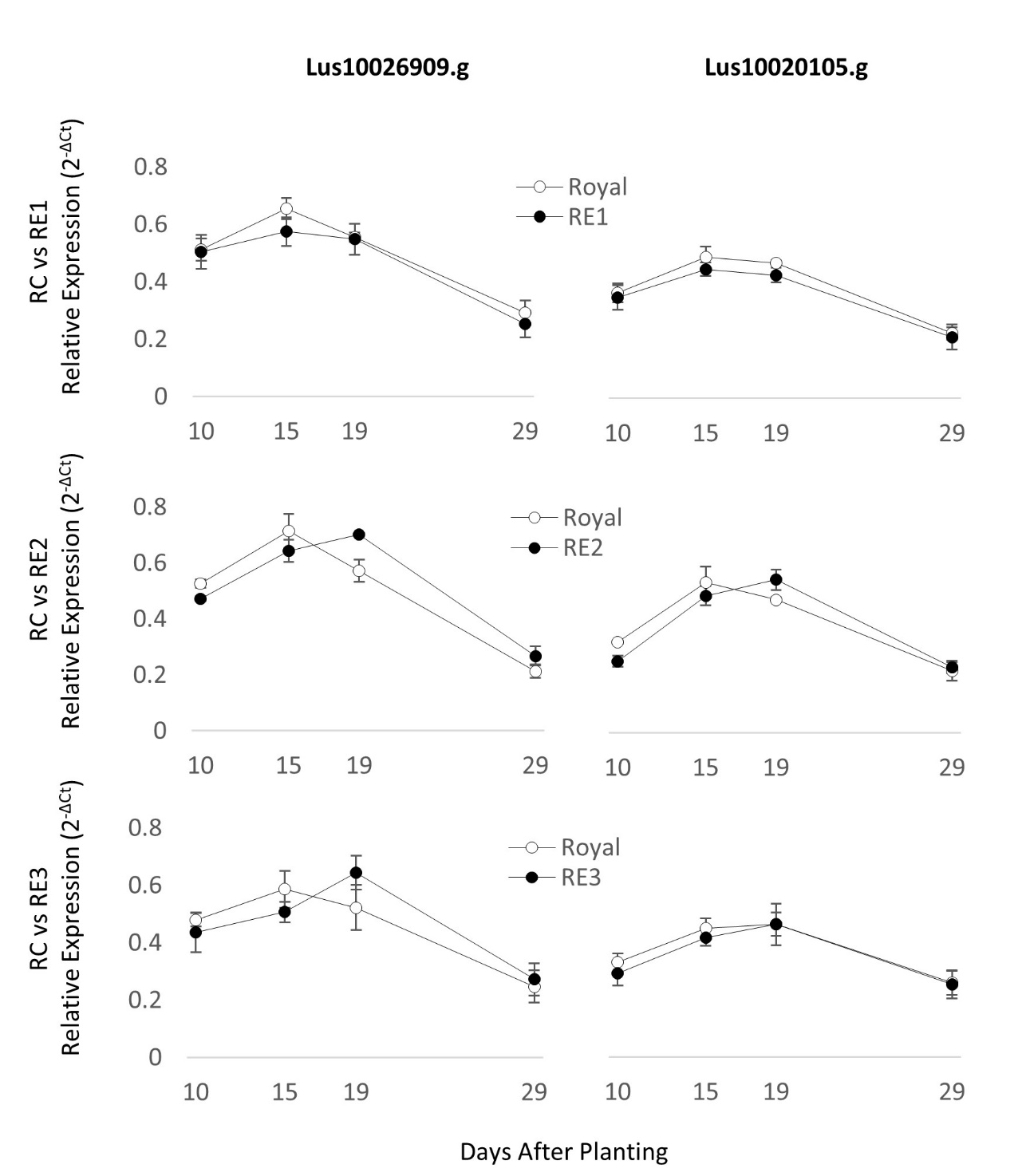


Relative expression of *CO* homologs Lus10026909 and Lus10020105 in RE1/2/3 and Royal leaves. RT-qPCR assays were performed in triplicate on cDNA from total RNA extracted from the third leaf, 10, 15, 19 and 29 days after planting. Three replications of the experiment were performed for each genotype. Error bars show standard errors.

Supplemental information: RT-qPCR 2^-∆Ct^ values

CO homolog expression

| **Lus10026909.g** | | | | | | | | |
| --- | --- | --- | --- | --- | --- | --- | --- | --- |
|  | **10 dap** | | **15 dap** | | **19 dap** | | **29 dap** | |
|  | **2^-∆Ct^** | **SE(2^-∆Ct^)** | **2^-∆Ct^** | **SE(2^-∆Ct^)** | **2^-∆Ct^** | **SE(2^-∆Ct^)** | **2^-∆Ct^** | **SE(2^-∆Ct^)** |
| Royal | 0.512398 | 0.03897 | 0.654197 | 0.036916 | 0.55525 | 0.016171 | 0.291299 | 0.042301 |
| RE1 | 0.503288 | 0.059192 | 0.574478 | 0.049747 | 0.547684 | 0.053289 | 0.252601 | 0.046367 |
| Royal | 0.525258 | 0.014356 | 0.713777 | 0.061341 | 0.571817 | 0.040337 | 0.21287 | 0.02476 |
| RE2 | 0.472107 | 0.008995 | 0.642733 | 0.040322 | 0.702234 | 0.00281 | 0.266962 | 0.035134 |
| Royal | 0.478598 | 0.02336 | 0.585855 | 0.063956 | 0.5215 | 0.078806 | 0.246477 | 0.055398 |
| RE3 | 0.434728 | 0.069393 | 0.50594 | 0.035745 | 0.643037 | 0.0593 | 0.27195 | 0.056141 |
| **Lus10020105.g** | | | | | | | | |
|  | **10 dap** | | **15 dap** | | **19 dap** | | **29 dap** | |
|  | **2^-∆Ct^** | **SE(2^-∆Ct^)** | **2^-∆Ct^** | **SE(2^-∆Ct^)** | **2^-∆Ct^** | **SE(2^-∆Ct^)** | **2^-∆Ct^** | **SE(2^-∆Ct^)** |
| Royal | 0.367913 | 0.031908 | 0.491293 | 0.036718 | 0.47212 | 0.009231 | 0.226535 | 0.021853 |
| RE1 | 0.35175 | 0.042707 | 0.449778 | 0.023628 | 0.429734 | 0.024867 | 0.213457 | 0.044227 |
| Royal | 0.315087 | 0.005769 | 0.523052 | 0.054921 | 0.46345 | 0.00854 | 0.215198 | 0.034899 |
| RE2 | 0.249093 | 0.019063 | 0.477467 | 0.033379 | 0.533482 | 0.035986 | 0.22743 | 0.019319 |
| Royal | 0.329747 | 0.031417 | 0.448358 | 0.035195 | 0.462087 | 0.072505 | 0.257959 | 0.041119 |
| RE3 | 0.290862 | 0.041619 | 0.41566 | 0.029058 | 0.462531 | 0.041126 | 0.25215 | 0.049004 |

|  |
| --- |

| FT homolog expression**Lus10004452.g** | | | | | | | | |
| --- | --- | --- | --- | --- | --- | --- | --- | --- |
|  | **10 dap** | | **15 dap** | | **19 dap** | | **29 dap** | |
|  | **2^-∆Ct^** | **SE(2^-∆Ct^)** | **2^-∆Ct^** | **SE(2^-∆Ct^)** | **2^-∆Ct^** | **SE(2^-∆Ct^)** | **2^-∆Ct^** | **SE(2^-∆Ct^)** |
| Royal |  |  | 1.44E-05 |  | 6.39E-06 | 4.42E-07 | 1.1E-05 | 5.29E-06 |
| RE1 |  |  | 0.000184 | 9.37E-06 | 0.000347 | 0.000216 | 0.000602 | 0.000272 |
| Royal |  |  |  |  | 1.59E-05 |  | 1.12E-05 | 8.51E-06 |
| RE2 | 1.47E-05 |  | 0.000249 | 1.19E-05 | 0.001141 | 0.000214 | 0.000892 | 0.00023 |
| Royal |  |  | 5.32E-06 |  | 1.46E-06 |  | 2.96E-06 | 1.37E-06 |
| RE3 | 3.89E-06 |  | 0.0001 | 7.49E-06 | 0.000316 | 0.000116 | 0.0002 | 9.46E-05 |
| **Lus10013532.g** | | | | | | | | |
|  | **10 dap** | | **15 dap** | | **19 dap** | | **29 dap** | |
|  | **2^-∆Ct^** | **SE(2^-∆Ct^)** | **2^-∆Ct^** | **SE(2^-∆Ct^)** | **2^-∆Ct^** | **SE(2^-∆Ct^)** | **2^-∆Ct^** | **SE(2^-∆Ct^)** |
| Royal |  |  |  |  |  |  | 1.18E-05 | 7.8E-06 |
| RE1 | 1.83E-06 |  | 9.25E-05 | 2.46E-05 | 0.000203 | 7.22E-05 | 0.000235 | 0.000105 |
| Royal |  |  |  |  |  |  | 2.48E-05 |  |
| RE2 |  |  | 0.000185 | 2.62E-05 | 0.000421 | 9.93E-05 | 0.000503 | 0.000154 |
| Royal |  |  |  |  | 1.78E-06 |  |  |  |
| RE3 |  |  | 5.96E-05 | 8.66E-06 | 0.000157 | 3.87E-05 | 0.000242 | 0.000126 |

GI homolog expression

| **5' end of Lus10028693.g** | | | | | | | | |
| --- | --- | --- | --- | --- | --- | --- | --- | --- |
|  | **10 dap** | | **15 dap** | | **19 dap** | | **29 dap** | |
|  | **2^-∆Ct^** | **SE(2^-∆Ct^)** | **2^-∆Ct^** | **SE(2^-∆Ct^)** | **2^-∆Ct^** | **SE(2^-∆Ct^)** | **2^-∆Ct^** | **SE(2^-∆Ct^)** |
| Royal | 0.081806 | 0.025043 | 0.155378 | 0.031536 | 0.154509 | 0.009848 | 0.135701 | 0.021542 |
| RE1 | 0.080863 | 0.018044 | 0.09715 | 0.007937 | 0.094764 | 0.006511 | 0.091155 | 0.017799 |
| Royal | 0.031517 | 0.017492 | 0.100336 | 0.026386 | 0.098436 | 0.010798 | 0.109926 | 0.019731 |
| RE2 | 0.035977 | 0.012816 | 0.070026 | 0.005109 | 0.07209 | 0.015156 | 0.076458 | 0.013684 |
| Royal | 0.075253 | 0.009331 | 0.12555 | 0.027751 | 0.142226 | 0.02232 | 0.116132 | 0.005878 |
| RE3 | 0.092565 | 0.010181 | 0.132069 | 0.028044 | 0.139242 | 0.030852 | 0.134579 | 0.01137 |
| **3' end of Lus10028693.g** | | | | | | | | |
|  | **10 dap** | | **15 dap** | | **19 dap** | | **29 dap** | |
|  | **2^-∆Ct^** | **SE(2^-∆Ct^)** | **2^-∆Ct^** | **SE(2^-∆Ct^)** | **2^-∆Ct^** | **SE(2^-∆Ct^)** | **2^-∆Ct^** | **SE(2^-∆Ct^)** |
| Royal | 0.09463 | 0.029489 | 0.182875 | 0.014656 | 0.248071 | 0.015058 | 0.199156 | 0.034028 |
| RE1 | 0.16144 | 0.01536 | 0.170325 | 0.014293 | 0.171999 | 0.011818 | 0.116229 | 0.011412 |
| Royal | 0.039717 | 0.024576 | 0.10739 | 0.031477 | 0.140148 | 0.012196 | 0.140301 | 0.026577 |
| RE2 | 0.045767 | 0.017071 | 0.124174 | 0.010795 | 0.133998 | 0.018978 | 0.11791 | 0.01237 |
| Royal | 0.078027 | 0.016179 | 0.15963 | 0.036038 | 0.172052 | 0.041329 | 0.157967 | 0.004242 |
| RE3 | 0.135837 | 0.015496 | 0.214 | 0.031902 | 0.212877 | 0.038023 | 0.187132 | 0.02393 |
| **Lus10028731.g** | | | | | | | | |
|  | **10 dap** | | **15 dap** | | **19 dap** | | **29 dap** | |
|  | **2^-∆Ct^** | **SE(2^-∆Ct^)** | **2^-∆Ct^** | **SE(2^-∆Ct^)** | **2^-∆Ct^** | **SE(2^-∆Ct^)** | **2^-∆Ct^** | **SE(2^-∆Ct^)** |
| Royal | 0.053681 | 0.009571 | 0.151912 | 0.088371 | 0.152399 | 0.065463 | 0.171645 | 0.021487 |
| RE1 | 0.064912 | 0.014738 | 0.114151 | 0.041213 | 0.162008 | 0.011591 | 0.038483 | 0.00469 |
| Royal | 0.027434 | 0.011919 | 0.080952 | 0.027024 | 0.076529 | 0.026071 | 0.081286 | 0.017459 |
| RE2 | 0.020927 | 0.010023 | 0.058078 | 0.01494 | 0.053484 | 0.028027 | 0.066058 | 0.021816 |
| Royal | 0.043494 | 0.00061 | 0.118378 | 0.024836 | 0.095936 | 0.02219 | 0.066089 | 0.004705 |
| RE3 | 0.097533 | 0.011926 | 0.078882 | 0.018609 | 0.081514 | 0.011849 | 0.065236 | 0.013225 |
